# Cetuximab-Containing Combinations in Locally Advanced and Recurrent or Metastatic Head and Neck Squamous Cell Carcinoma

**DOI:** 10.3389/fonc.2019.00383

**Published:** 2019-05-20

**Authors:** Miren Taberna, Marc Oliva, Ricard Mesía

**Affiliations:** ^1^Medical Oncology Department, Catalan Institute of Oncology, ONCOBELL, Bellvitge Biomedical Research Institute (IDIBELL), L'Hospitalet de Llobregat, Barcelona, Spain; ^2^Medicine Department, Barcelona University, Barcelona, Spain; ^3^Division of Medical Oncology and Haematology, Princess Margaret Cancer Centre, University of Toronto, Toronto, ON, Canada; ^4^B-ARGO Group, Medical Oncology Department, Catalan Institute of Oncology, Badalona, Spain

**Keywords:** head and neck cancer, head and neck squamous cell carcinoma, HPV-positive head and neck cancer, head and neck cancer treatment, anti-EGFR therapy, cetuximab

## Abstract

Cetuximab remains to date the only targeted therapy approved for the treatment of head and neck squamous cell carcinoma (HNSCC). The EGFR pathway plays a key role in the tumorigenesis and progression of this disease as well as in the resistance to radiotherapy (RT). While several anti-EGFR agents have been tested in HNSCC, cetuximab, an IgG1 subclass monoclonal antibody against EGFR, is the only drug with proven efficacy for the treatment of both locoregionally-advanced (LA) and recurrent/metastatic (R/M) disease. The addition of cetuximab to radiotherapy is a validated treatment option in LA-HNSCC. However, its use has been limited to patients who are considered unfit for standard of care chemoradiotherapy (CRT) with single agent cisplatin given the lack of direct comparison of these two regimens in randomized phase III trials and the inferiority suggested by metanalysis and phase II studies. The current use of cetuximab in HNSCC is about to change given the recent results from randomized prospective clinical trials in both the LA and R/M setting. Two phase III studies evaluating RT-cetuximab vs. CRT in Human Papillomavirus (HPV)-positive LA oropharyngeal squamous cell carcinoma (De-ESCALaTE and RTOG 1016) showed inferior overall survival and progression-free survival for RT-cetuximab combination, and therefore CRT with cisplatin remains the standard of care in this disease. In the R/M HNSCC, the EXTREME regimen has been the standard of care as first-line treatment for the past 10 years. However, the results from the KEYNOTE-048 study will likely position the anti-PD-1 agent pembrolizumab as the new first line treatment either alone or in combination with chemotherapy in this setting based on PD-L1 status. Interestingly, cetuximab-mediated immunogenicity through antibody dependent cell cytotoxicity (ADCC) has encouraged the evaluation of combined approaches with immune-checkpoint inhibitors in both LA and R/M-HNSCC settings. This article reviews the accumulated evidence on the role of cetuximab in HNSCC in the past decade, offering an overview of its current impact in the treatment of LA and R/M-HNSCC disease and its potential use in the era of immunotherapy.

## Introduction

The role of the epidermal growth factor receptor (EGFR) in the development and progression of head and neck squamous cell carcinoma (HNSCC) has been widely studied (1). EGFR is a transmembrane glycoprotein member of the tyrosine kinase growth factor receptor family that regulates cell growth and proliferation (2). This receptor is overexpressed in up to 90% of HNSCC and has been associated with decreased survival (2–4). The accumulating evidence led to the evaluation of agents targeting the EGFR pathway in this tumor type.

Cetuximab is the only anti-EGFR agent that has been proven effective for the treatment of HNSCC thus far (5, 6). Cetuximab is a chimeric IgG1-subclass monoclonal antibody that binds to the extracellular domain of the EGFR with higher affinity than the natural ligands EGF and TGFα, blocking the activation of its intracellular domain and subsequent tyrosine kinase-dependent signal transduction pathway (7). Cetuximab also stimulates the internalization of EGFR, removing the receptor from the cell surface and thus preventing its interaction with the ligand (8). Additionally, as an IgG1 molecule, it stimulates antibody dependent cell cytotoxicity (ADCC) (9, 10). Several preclinical studies demonstrated that EGFR inhibition by cetuximab increases the efficacy of radiotherapy (RT) (11) since it decreases the proportion of cells in S phase and increases that of G1 phase, facilitates apoptosis, decreases the capacity of DNA repair, and has an antiangiogenic effect (12, 13). Moreover, cetuximab enhanced the antitumor activity of several chemotherapeutic drugs in mouse xenograft models (14).

Cetuximab reached the clinics a decade ago at a time where treatment options for HNSCC were very limited. Chemo-RT (CRT) or RT alone depending on patients' functional status and comorbidities were the only available conservative treatment options in the locally-advanced (LA) setting. Cetuximab improved the variability of choice (5) although the clinical practice finally positioned its use in combination with RT (RT-Cx) to those patients unfit to receive high dose cisplatin or those who had previously received three cycles of cisplatin-based induction chemotherapy (ICT) and had significant residual toxicity. In recurrent/metastatic (R/M) HNSCC, we had to choose between monotherapy and polychemotherapy until the results from the EXTREME trial. The addition of cetuximab to first-line chemotherapy significantly improved disease control and overall survival (OS) when compared to chemotherapy alone becoming the new standard of care in this patient population (6). However, despite the EXTREME regimen has remained the recommended first-line as per the clinical guidelines for the past 10 years, its use has been limited outside Europe. Nevertheless, the results of the KEYNOTE-048 clinical trial (NCT02358031) evaluating the activity of pembrolizumab (anti-PD-1 therapy) with or without chemotherapy will likely lead immunotherapy to the first line treatment for the majority of R/M HNSCC patients (15).

Besides cetuximab, several anti-EGFR monoclonal antibodies have been tested in HNSCC, including panitumumab, zalutumumab and nimozutumab (1, 16–18). Among all these, panitumumab is the only one that has been evaluated in randomized phase III clinical trials in both LA and R/M disease, failing to show any improvement in LRC or survival when compared to the standard of care (16, 19). Some authors argued that, unlike cetuximab (IgG1), the inability of panitumumab (IgG2) to produce antitumor activity through ADCC and natural killer (NK) cell activation might have explained the lack of benefit from this agent in HNSCC (7, 20). To date, cetuximab is the only anti-EGFR antibody with proven efficacy and survival gain in HNSCC.

In this article, the authors review the evidence accumulated on the role of cetuximab in HNSCC in the past decade, offering an overview of its current impact in the treatment of LA and R/M disease and its potential use in the era of immunotherapy.

## Locally-Advanced Head And Neck Squamous Cell Carcinoma

### Has Cetuximab Reached a Plateau in the Treatment of LA-HNSCC?

Cetuximab is the only targeted therapy that has been proven effective for the treatment of LA-HNSCC (5). The implications of EGFR overexpression in resistance to RT has been reported in several studies (2, 13). Preclinical models showed that EGFR blockade by cetuximab increases radiation-induced apoptosis and blocks secondary repair mechanisms dependent on PI3K/AKT/MAPK and JAK/STAT3 downstream signaling pathways, indicating a synergistic effect of the RT-Cx combination (21, 22). In 2006, the Bonner randomized phase III study evaluated the addition of cetuximab to RT in over 400 patients with LA-HNSCC showing a significant improvement in locoregional control (LRC) (24.4 vs. 14.9 months, *p* = 0.005) and OS (49 vs. 29 months, *p* = 0.006) with the combination (5). These results led to the FDA approval of cetuximab for the treatment of LA-HNSCC and RT-Cx was incorporated in the clinical guidelines as a validated alternative to standard chemoradiotherapy (CRT) in this setting (23, 24).

The survival benefit obtained by the addition of cetuximab to RT was confirmed by the 5-year update of the Bonner trial (5-year OS of 45.6% for the combination vs. 36.4% for RT alone, *p* = 0.018). However, the lack of a direct comparison with standard of care CRT in randomized phase III trials and the differential toxicity profile of both drugs contributed to limit the use of RT-Cx to patients considered “unfit” for cisplatin-based CRT despite this patient population was not represented in the Bonner trial (25, 26). Whether both treatments are equivalent in terms of efficacy has remained unclear over the years as several retrospective series and meta-analysis had showed mixed results (27–30). The meta-analysis conducted by Huang et colleagues in 2016 including up to 31 studies and over 4,000 patients showed no differences in disease control or survival beyond the 2-year threshold between both treatment combinations, although the overall pooled HR for OS, progression-free survival (PFS) and LRC were significantly inferior in the arm of RT-Cx (31). However, the intrinsic limitations of the retrospective analyses including unmatched patient characteristics and biased treatment selection based on patient's baseline condition difficulted the interpretation of these data. The prospective randomized phase II trial evaluating CRT vs. RT-Cx conducted by Magrini et al. failed to show any significant differences in treatment outcome between both arms, despite the 2-year LRC and 2-year cancer specific survival rates were lower among patients treated with RT-Cx (53 vs. 80%; and 68 vs. 81%, respectively) (32). Since the study was stopped prematurely, with only 35 patients per arm, it was underpowered for its primary endpoint, hence definitive conclusions could not be drawn from its results. In HPV-positive LA oropharyngeal cancer (OPC), two randomized phase III studies evaluating RT-Cx vs. CRT (CDDP) in HPV-positive LA-OPC (De-ESCALAaTE and RTOG 1016) have recently reported significantly worse survival and disease control rates in the RT-Cx arm (33, 34). A phase III randomized prospective study comparing RT-Cx vs. CRT in LA-HNSCC with OS as primary endpoint is currently on-going and might provide a more definitive answer (NCT01969877).

The positive results obtained by the addition of cetuximab to platinum-based chemotherapy in the first line R/M HNSCC led to its evaluation in combination with CRT and ICT in the LA setting (35–39). Few publications have reviewed the studies conducted to date indicating that intensification therapy with cetuximab given concurrently with CRT does not seem to improve patient outcome but adds significant toxicity (1, 40, 41). The only phase III randomized trial evaluating cetuximab plus standard CRT with single agent cisplatin vs. CRT failed to show any improvement in LRC, distant control nor survival in the cetuximab arm but did show higher rate of grade 3/4 toxicity (36). Recently, the GORTEC 2007-01 phase III study that evaluated RT-Cx plus carboplatin and 5-FU vs. RT-Cx alone showed no OS benefit despite better PFS and LRC, with again significantly grade 3–4 toxicity increment (42).

The addition of cetuximab to different ICT regimens appeared to improve response rates and extend survival when compared to historical controls, especially when combined with taxane-based chemotherapy regimens (43–45). The role of ICT in LA-HNSCC has been widely debated since it has not demonstrated a sustained survival benefit when compared to standard CRT in randomized trials (44, 46–49). Overall, the lack of control arms allowing direct comparison in the studies evaluating cetuximab-based ICT combinations and the severe toxicity increased in some of the trials, particularly when using the TPF regimen, has precluded a widespread use of this treatment modality among the head and neck community (49–51). However, RT-Cx given sequentially to ICT does seem to offer similar results in terms of efficacy when compared to standard CRT, with an overall acceptable toxicity, which is particularly relevant in patients who previously received cisplatin as part of the ICT (37, 39, 52, 53).

To date, no randomized phase III trials have evaluated the role of cetuximab vs. cisplatin in the adjuvant treatment of resected LA-HNSCC. The phase II study RTOG-0234 did investigate the addition of cetuximab to weekly docetaxel or cisplatin and RT in patients with resected HNSCC and high risk features (positive margins and/or extranodal extension) (54). Despite both regimens were tolerable, and the combination with docetaxel showed promising disease-free survival, these regimens were never compared against standard post-operative high-dose cisplatin and RT in a randomized study, and therefore its use was not widespread. Similarly, the ACCRA-HN phase 2 study compared post-operative RT-Cx vs. RT-Cx plus cisplatin and 5-FU (NCT00791141), although the results of these study have not been published yet.

Overall, with the current available data, RT-Cx remains a valid treatment option for the treatment of LA-HNSCC, although standard of care CRT (cisplatin 100 mg/m^2^ every 3 weeks) should be pursued when feasible. Sequential RT-Cx following ICT as part of organ-preservation strategy is a reasonable alternative to avoid acute and late toxicity, but other treatment combinations should be avoided. There is no evidence to support the use of cetuximab in the adjuvant setting.

### Other Cetuximab Containing Combinations in LA-HNSCC

Cetuximab has also been investigated in combination with a variety of chemotherapy agents and targeted therapies in multiple clinical trials for LA-HNSCC although none of them has reached the clinics yet. Based on the good results observed in combination with taxanes in the R/M setting and within ICT regimens in the LA disease above mentioned, a few trials evaluated the combination of cetuximab with taxanes concurrent with RT. A phase I/II study investigated nab-paclitaxel plus cetuximab and low-dose cisplatin (20 mg/m^2^) showing similar 2-year PFS compared to historical controls (60%) and tolerable toxicity, but no further evaluation of this regimen is on-going (55). A separate phase II randomized study is evaluating docetaxel plus cetuximab concurrent with RT vs. standard CRT, but results are yet to be presented (NCT02128906). Other chemotherapy combinations, such as pemetrexed plus cetuximab and RT have also been tested in phase II studies with similar efficacy and tolerability, but have not been further investigated in phase III randomized trials (56). In regards to targeted therapies, Bevacizumab, an anti-VEGF monoclonal antibody, has been investigated in combination with cetuximab in the LA-HNSCC based on preclinical data suggesting a key role for VEGF pathway in the resistance to RT and Cetuximab (57, 58). Given the promising activity and tolerability seen in early studies performed in the R/M setting, two phase II studies evaluated bevacizumab in combination with RT plus pemetrexed and RT plus cisplatin (59, 60). Despite positive results in terms of efficacy, the increased toxicity and the lack of comparative arms precluded further investigation of bevacizumab in this setting. Other antiangiogenic agents, such as sunitinib, have been combined with cetuximab (NCT00906360) but results are still pending.

The inhibition of other molecular targets including the Src family kinase, the Poly (ADP-Ribose) Polymerase (PARP), Cyclin Dependent Kinase complex (CDK) has shown to have a synergistic effect in combination with EGFR blockade by cetuximab and overcome resistance to this agent according to several studies using preclinical models (61–64). Dasatinib (SRC inhibitor), olaparib (PARP inhibitor), and pablociclib (selective CDK 4/6 Inhibitors) are currently subject of investigation in combination with cetuximab and RT in the LA setting (NCT00882583, NCT01758731, NCT03024489, respectively). Despite preliminary results from early trials have showed a safe toxicity profile with the combination, their efficacy is yet to be determined (65, 66). Noteworthy, preclinical studies using xenograft models suggested that dasatinib might be detrimental for tumor control when combined with cetuximab and RT (61). Therefore, we must remain cautious while awaiting the results from the ongoing clinical trials.

A summary of published phase II/III studies evaluating cetuximab combinations in LA-HNSCC is provided (Supplementary Table 1).

### Patient Selection: Are the Bonner Trial Results Reproducible in Daily Practice?

Besides the severity of cetuximab-induced skin rash no other biomarkers have shown to predict clinical activity of cetuximab (67). Several biological and molecular candidates have been tested including EGFR protein expression, truncated receptor variants, such as EGFRvIII, or mutations at the level of EGFR gene or downstream, such as KRAS, but thus far none of them has been proven effective in predicting response (or resistance) to cetuximab in HNSCC (68–71). Therefore, treatment selection between standard CRT and RT-Cx in patients with LA-HNSCC has been often based on patient baseline condition and comorbidities, taking into consideration the differential toxicity profile between cetuximab and cisplatin. Patients with significant comorbidities and/or poor ECOG performance status and the elderly are usually ineligible for cisplatin and as such, they tend to be treated with cetuximab (72). Cetuximab's acute side effects mainly include infusion reactions, skin rash and mucositis, with no major organ-specific or chronic toxicity described, making it a suitable option for this patient population (29). However, the majority of patients enrolled in the Bonner study were under 70 years old, with no significant comorbidities and a Karnofsky index ≥80 (5). In this regard, an exploratory *post-hoc* analysis published in the 5-year update of the Bonner trial suggested that younger patients with good performance status were more likely to benefit from this combination (25). Several studies have reported increased risk of local and systemic toxicity from cetuximab in patients at older age, with significant baseline comorbidities or with poor performance status, including cytopenia, bloodstream infections and sepsis (73). Some authors have postulated that fragile patients might be more susceptible to toxicity due to local and systemic inflammatory responses triggered by cetuximab-induced antibody-dependent cellular cytotoxicity (74).

Altogether these data suggest that the expected efficacy and toxicity from RT-Cx might differ when compared to the Bonner trial in our daily practice given our biased patient selection for this treatment. Hence, the need for prospective trials focusing on this frail population is timely.

## Recurrent Or Metastatic Head And Neck Squamous Cell Carcinoma

### Cetuximab in R/M HNSCC

In 2006, a phase I/II study investigating cetuximab in combination with cisplatin/carboplatin and 5-FU in R/M HNSCC showed promising activity and acceptable tolerability (75). The subsequent phase III randomized study evaluating the addition of cetuximab to cisplatin/carboplatin and 5-FU for a total of 6 cycles followed by maintenance cetuximab (EXTREME regimen) vs. chemotherapy alone in the first-line R/M setting conducted by Vermorken and colleagues demonstrated the superiority of the combination in terms of OS and response rate (6). The combined regimen improved both OS and PFS from 7.4 to 10.1 months; and from 3.3 to 5.6 months, respectively, when compared to chemotherapy alone. The overall response rate (ORR) was also increased from 20 to 36% with the combination. The most common grade 3 or 4 adverse events in the chemotherapy-alone and cetuximab groups were anemia (19 and 13%, respectively), neutropenia (23 and 22%), and thrombocytopenia (11% in both groups). Of 219 patients receiving cetuximab, 9% had grade 3 skin reactions (6). The results from this study set the EXTREME regimen as the new standard of care for the first-line treatment of R/M HNSCC (24, 76), which has remained unchanged since 2008. Noteworthy, subsequent observational studies (SOCCER, DIRECT, ENCORE) endorsed the results from the EXTREME study in the daily clinical practice (76–78). In addition, about 14% of the patients treated with the EXTREME regimen have been reported to have long-term responses (35).

**Figure 1:**
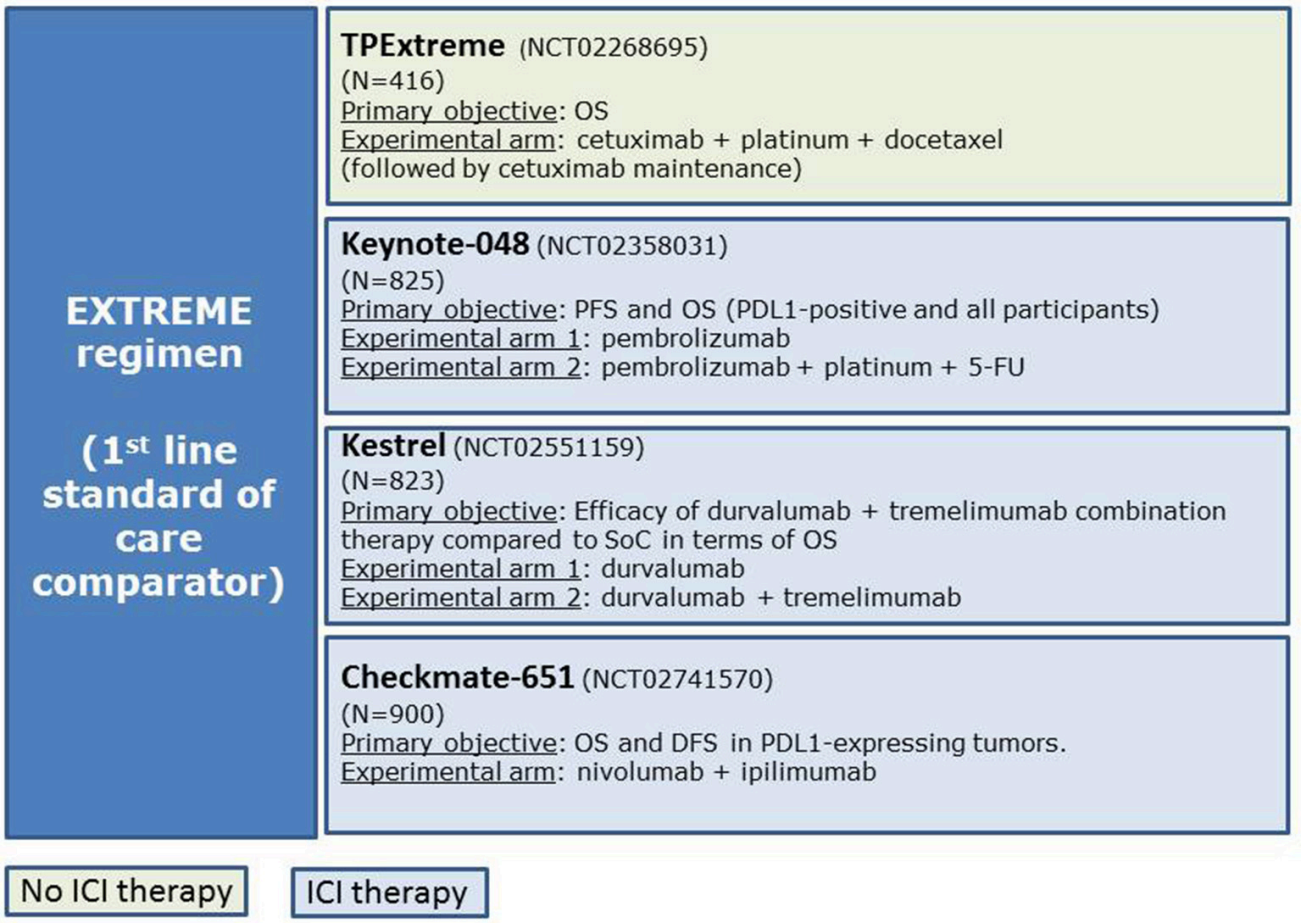
Main clinical trials evaluating the efficacy of new schemes of treatments compared with the EXTREME regimen.

Several randomized trials are currently evaluating immune-checkpoint inhibitors (ICI) alone or in combination with chemotherapy against the EXTREME regimen in an attempt to improve patients' survival and quality of life. The main phase III randomized clinical trials are keynote-048 (NCT02358031), Kestrel (NCT02551159), and Checkmate-651 (NCT02741570) (Figure 1).

The preliminary results of the Keynote 048 trial have been recently presented. This phase III study evaluated the efficacy of pembrolizumab (anti-PD-1) alone and in combination with cisplatin/carboplatin plus 5FU vs. the EXTREME regimen as first-line therapy for R/M-HNSCC based on PD-L1 expression by CPS (combined positive score) (15). The study showed better OS in the pembrolizumab monotherapy arm vs. EXTREME when PD-L1 expression ≥1 and ≥20% by CPS (HR 0.78 [0.64–0.96], *p* = 0.0086 and HR 0.61 [0.45–0.83], *p* = 0007, respectively) and in the pembrolizumab plus chemotherapy arm vs. EXTREME regardless of PD-L1 expression (10.7 vs. 13 months, HR 0.77 IC 95% 0.63–0.93, *p* = 0.0034). With these results, pembrolizumab monotherapy and the combination of pembrolizumab-chemotherapy will likely become the new first line treatment for R/M-HNSCC based on CPS PD-L1 expression. However, the complete results of the study are still to be published, and full biomarker analyses are awaited.

### Improving the EXTREME Regimen With Other Chemotherapy Agents

Within the 3 drugs of the EXTREME regimen, 5-FU is the most difficult one to be administered in terms of logistics, as it requires 24-h continuous infusion for a total of 4 days. Furthermore, 5-FU is associated with increased rate of mucositis and diarrhea, and its use is not recommended in patients with cardiovascular diseases or with dihydropyrimidine dehydrogenase deficiency. Therefore, the substitution of 5-FU with a taxane is being investigated as a potentially new scheme for R/M-HNSCC. Preclinical data have suggested a synergistic effect when combining taxanes with cetuximab (79). Bossi et al. demonstrated in a phase IIb clinical trial (B409) that the cetuximab-cisplatin regimen was non-inferior to the cetuximab-cisplatin-paclitaxel regimen in terms of PFS [HR for cetuximab-cisplatin vs. cetuximab-cisplatin-paclitaxel [0.99; 95% CI: 0.72–1.36, *P* = 0.906; margin of non-inferiority (90% CI of 1.4) not reached] (80). Interestingly, the ORR achieved by the three drugs regimen was >50%. Grade 4 toxicities were reported in 14% of patients receiving cetuximab-cisplatin and 33% of those receiving cetuximab-cisplatin-paclitaxel (*P* = 0.015), but by substituting 5-FU for paclitaxel, the rates of grade_3 cardiac toxicity appeared lower in both arms and no sepsis was described compared to EXTREME regimen (80). Argiris et al. introduced for the first time the combination of cisplatin-docetaxel-cetuximab in a phase II clinical trial for LA-HNSCC (81). The GORTEC group developed this combination (named “TPEx”) in a phase II study (GORTEC 2008-03) for R/M disease (82). They demonstrated that 4 cycles of docetaxel combined with cisplatin (75 mg/m^2^ both at day 1) and weekly cetuximab (250 mg/m^2^) followed by maintenance cetuximab (500 mg/m^2^, every 2 weeks) were feasible, active, and with a manageable safety profile in fit patients with R/M HNSCC. ORR at week 12 was 44.4%; median OS and PFS were 14.0 and 6.2 months, respectively. In addition, the ORR increased to, 16.8 and 7.1 months in the population of patients with disease control after the initial 4 cycles of complete TPEx regimen. The European TPEx randomized phase II study evaluating the TPEx regimen vs. the EXTREME regimen is currently ongoing and will contribute in determining which one might be the best treatment option for the first-line treatment in this patient population (NCT02268695). Other taxane-based combinations in first-line R/M-HNSCC are also being currently evaluated, such as the phase II study CACTUX trial investigating nab-paclitaxel and cetuximab (NCT02270814).

### From the Clinical Trial to an Outpatient Clinic: Treatment for Unfit Patients.

In daily clinical practice, a considerable number of patients with HNSCC have significant comorbidities and/or a frail functional status that makes them unfit to receive the EXTREME regimen. This patient population is usually underrepresented in clinical trials. Despite the lack of prospective randomized data, the combination of taxanes with cetuximab or a single agent (paclitaxel, docetaxel, cetuximab, methotrexate, 5-FU, capecitabine…) have been suggested as alternative treatment options for these patients. (83). The combination of docetaxel/paclitaxel with cetuximab appears to have a manageable safety profile and good response rates. Few prospective single-arm phase II studies have investigated this combination: the first study was conducted by Hitt et al. and evaluated cetuximab plus paclitaxel as first-line treatment showing an ORR of 54% (95% CI: 39–69) (84). Interestingly, 61% of the population included in the trial had a Karnofky Index of 70–80%. The Knoedler et al. study evaluated cetuximab plus docetaxel in patients who failed a platinum-based therapy, achieving an overall disease control rate of 51% (85). Recently, a retrospective study showed that the combination of paclitaxel and cetuximab could be a suitable treatment option in HNSCC patients with platinum-based CRT-refractory disease (86).

In addition, based on the keynote 048 preliminary results (15), pembrolizumab monotherapy might represent an option in patients unfit for cisplatin-based chemotherapy.

### Cetuximab Containing Combinations in R/M HNSCC

The combination of cetuximab with different chemotherapy regimens and with other targeted agents against key pathways involved in HNSCC tumorigenesis and progression has been investigated in several clinical trials.

Besides the EXTREME regimen and taxane-based chemotherapy combinations, cetuximab has been also been evaluated in combination with other chemotherapies, such as pemetrexed or methotrexate. A phase III study comparing pemetrexed plus cisplatin vs. cisplatin alone in R/M HNSCC did not significantly improve survival for the intent-to-treat population (87). Despite this result, a phase II study evaluated the addition of cetuximab to this regimen. However, the study did not reach its primary end-point (PFS) and was considered negative (88). The Dutch Head and Neck Society is currently investigating cetuximab in combination with methotrexate in a Phase Ib-II study (NCT02054442).

Phosphatidylinositol 3-kinase (PI3-K) inhibitors were one of the most promising targeted therapies for cetuximab-based combinations given the relevance of the PI3K pathway in proliferation, apoptosis and cell differentiation of HNSCC. Two phase Ib/II studies are investigating the combinations of cetuximab and PI3K inhibitors, the first one with BKM 120 (NCT01816984), and the second one with BYL719 (NCT01602315). A randomized phase II study evaluated the addition of PX-866 to cetuximab in patients with advanced R/M-HNSCC; PX-866 addition did not show any significant improvement in PFS nor OS (89).

Cilengitide, an integrin inhibitor, has also been investigated in the ADVANTAGE phase I/II study. The phase II part was a multicenter, open-label, randomized and controlled study investigating cilengitide 2,000 mg once or twice weekly plus chemotherapy based on EXTREME regimen vs. EXTREME regime alone. Neither of the cilengitide-containing regimens demonstrated a PFS benefit over EXTREME regimen alone in R/M-SCCHN patients (90).

Preclinical studies had also suggested that mammalian target of rapamycin (mTOR) inhibitors might overcome the resistance to EGFR blockade and augment cetuximab efficacy. The combination of everolimus (RAD001) with cetuximab and carboplatin was explored in a phase I study showing encouraging antitumor activity in a selected group of patients (91). The currently on-going MAESTRO study is evaluating temsirolimus with or without cetuximab for previously treated R/M-HNSCC patient (NCT01256385).

Based on pre-clinical data, Argiris et al. conducted a phase II study to evaluate the efficacy of bevacizumab and cetuximab in patients with R/M SCCHN refractory to first-line treatment. The modest median PFS and OS (2.8 and 7.5 months, respectively) did no lead to further development of this regimen (59).

Other agents, such as patritumab (U3-1287), an anti-HER3 monoclonal antibody, in combination with platinum-based therapy and cetuximab has been studied in a double-blind phase 2 study, but no results have been released yet (NCT02633800). Cyclin-dependent-kinase-inhibitors, such as palbociclib are also been tested in combination with avelumab and cetuximab for R/M-HNSCC (NCT03498378).

A summary of published phase II/III studies evaluating cetuximab combinations in RM-HNSCC is provided (Supplementary Table 2).

## Cetuximab In HPV-Positive Opc

HPV-positive OPC represents a biologically distinct disease characterized by increased radiosensitivity and improved overall survival when compared to HPV-negative OPC (92, 93). Retrospective subgroup analyses from randomized trials had reported better outcome in patients with HPV-positive disease, regardless of treatment (94–96). Given the acute and potential long-term side-effects associated to CRT (97), many on-going clinical trials are currently evaluating de-escalation treatment strategies to reduce long-term toxicity without compromising survival in this subgroup of patients (98). Chemo-sparing approaches to replace cisplatin by other agents, such as cetuximab or immune checkpoint inhibitors (ICI) given concurrent with radiation are the most attractive options (NCT02254278, NCT01874171, NCT03410615). Main de-escalation clinical trials ongoing evaluating cetuximab in combination with RT are summarized on Table 1.

**Table 1:** Main de-escalation clinical trials ongoing evaluating cetuximab in combination with RT for HPV-related OPSCC.

**Strategy**	**Country**	**Trial**	**Phase**	***N***	**HPV diagnosis technic**	**Primary objective**	**Comments**
Cetuximab with IMRT radiation (in comparison with IMRT-cisplatin)	US	RTOG 1016 NCT01302834	Phase III	987	p16^INK4a^ IHC	OS (non-inferiority)	Cisplatin day 1 and 22
	Australia	TROG 1201 NCT01855451	Phase III	189	p16^INK4a^ IHC	Symptom severity	Weekly cisplatin Evaluate smoking history
	UK	De-ESCALaTE NCT01874171	Phase III	334	p16^INK4a^ IHC	Overall severe (acute and late) toxicity (Grade 3–5)	Cisplatin day 1, 22, and 43 Bulky disease with >10 p/y smoking history excluded

The role of cetuximab in HPV-positive OPC has been extensively debated (99, 100). The exploratory subgroup analysis from the 5-year survival update of the Bonner study seemed to favor the use of cetuximab in young patients (<65 years old), with primary OPC and high Karnofsky index (25). The *post-hoc* analysis published by Rosenthal et al. evaluating the differential effect of RT-Cx in p16-positive vs. p16-negative patients treated within the Bonner trial showed higher OS gain in the p16-positive subgroup (HR 0.38 vs. 0.93, respectively) (101). However, no significant interaction was observed between p16 positivity and treatment effect. Similarly, the exploratory subgroup analysis from the EXTREME trial in the recurrent/metastatic setting reported increased survival in HPV-positive vs. HPV-negative patients (102). Conversely, in the CONCERT-2 and SPECTRUM clinical trials evaluating panitumumab in the LA and R/M setting, respectively, patients with p16-positive tumors had significantly lower survival when compared to p16-negative disease (16, 45). The fact that both studies were negative for their primary endpoints and that the threshold used for p16 positivity was lower than the standard recommendations (10% staining instead of 70%) made interpretation of these results difficult.

The accumulating evidence on the biological rationale behind the use of cetuximab in HPV-positive disease had been inconsistent with the abovementioned subgroup analysis. Several studies had highlighted the absence of EGFR protein overexpression and EGFR/HER pathway activation in HPV-driven tumors (103–106). Moreover, a comprehensive analysis of the genomic landscapes of HPV-positive and negative HNSCC confirmed the lack of EGFR aberrations in HPV-positive tumors and an increased frequency of RAS mutations when compared to HPV-negative tumors (107). Noteworthy, anti-EGFR therapies are not currently recommended for treatment of anogenital HPV-positive cancer (108, 109) highlighting the lack of sense of targeting EGFR in HPV-related tumors.

In concordance with these data, latter studies did show decreased efficacy of RT-Cx in HPV-positive disease (27, 110). The interim subgroup analysis from a prospective phase II trial evaluating RT-cetuximab vs. CRT with weekly cisplatin in LA-HNSCC showed a trend favoring the cisplatin arm in all outcome parameters including LRC, PFS and OS in the p16-positive group (NCT01216020) (110). Unfortunately, this study was terminated due to slow recruitment and the sample was limited and therefore unpowered to show significant differences. Summarized clinical data investigating anti-EGFR therapies on HPV-positive OPC are presented on Table 2. It is important to highlight that most of these studies based the HPV positivity on p16 staining exclusively. Recently published data suggest that p16 expression alone may not be accurate to classify OPC as HPV-positive, and other biomarkers, such as HPV DNA might be required to characterize these tumors (117–119).

**Table 2:** Summarized of clinical data investigating anti-EGFR therapy on HPV-positive HNSCC.

**References (Study)**	**Treatment**	**HPV positivity analysis**	**Result**
**Recurrent and metastatic HNSCC**			
Vermorken et al. (45) (SPECTRUM)	Cisplatin and fluorouracil ± panitumumab	Prospective	Addition of panitumumab to cisplatin-based chemotherapy significantly improves OS and PFS only in HPV negative HNSCC patients
Vermorken et al. (90) (EXTREME)	Cisplatin and fluorouracil ± cetuximab	Retrospective	Survival benefit of adding cetuximab to platinum-based chemotherapy was independent of p16 status
Fayette et al. (111) (MEHGAN)	Cetuximab vs. cetuximab- duligotuzumab.	Prospective	HPV-negative HNSCC but not HPV-positive are most likely to respond to EGFR blockage by cetuximab or duligotuzumab.
Seiwert et al. (112) (BIBW 2992 trial)	Afatinib vs. cetuximab	Prospective	HPV positive HNSCC patients had a lower response rate to EGFR inhibitors compared with HPV negative patients
**Locally advanced HNSCC**			
Pajares et al. (113) (Restrospective series)	Cisplatin-RT vs. cetuximab-RT	Retrospective series	p16-positive patients may benefit more from RT combined with EGFR inhibitors than with cisplatin
Koutcher et al. (114) (Retrospective series)	Cisplatin-RT vs. cetuximab-RT	Retrospective series	Treatment with cisplatin not cetuximab predict for better OS, FFS and locoregional control
Ang et al. (36) (RTOG 0522 study)	Cisplatin ± cetuximab with AFX RT	Prospective	The addition of cetuximab produce no benefit in PFS or OS in patient with p16 positive or negative HNSCC
Rosenthal et al. (101) (IMCL-9815 phase III Study)	RT vs. cetuximab-RT	Retrospective	Better outcomes in both groups p16-positive and p16-negative when treated with cetuximab and RT in comparison with RT alone
Mesía et al. (115) (CONCERT-1)	Cisplatin-RDT ± panitumumab	Prospective	No benefit was noted with the addition of panitumumab in either PFS or OS in the patients with p16-postive tumors
Giralt et al. (16) (CONCERT-2)	Panitumumab-RT vs. cisplatin-RT	Prospective	Better outcomes for cisplatin-RT (few p16 positive patients included)
Ou et al. (116) (Retrospective series)	Cisplatin-RT vs. cetuximab-RT	Restrospective series	Better outcomes in patients receiving concurrent cisplatin over cetuximab regardless of HPV/p16 status
Mena et al. (117) (Retrospective series)	Cisplatin-RT vs. cetuximab-RT vs. surgery/RT vs. ICT/RT	Retrospective series	Improved OS for all treatment schemes with the exception of those who underwent cetuximab-RT

*AFX RT, Accelerated fractionation radiotherapy; HNSCC, Head and neck squamous cell carcinoma; FFS, failure free survival; OS, overall survival; PFS, progression free survival; RT, radiotherapy*.

The results from three de-escalation randomized phase III clinical trials (Table 1) evaluating RT-Cx vs. standard CRT with cisplatin provided a definitive answer regarding the role of cetuximab in HPV-positive OPC patients. The RTOG 1016, a phase III non-inferiority study showed inferior OS in the RT-Cx arm [5 years OS 84.6 (95% CI 73.4–82.5) vs. 77.9% (95% CI 73.4–82.5)] (34). The De-SCALaTE phase III clinical trial revealed the same rate of severe and all-grade toxicities when compared to CRT and worse OS in the RT-Cx arm (2 years OS 97.5 vs. 89.4%; HR = 4.99; 95% CI: 1.70–14.67 (33). Therefore, CRT will remain the standard of care for HPV-positive LA-OPC while awaiting results from other on-going de-escalation clinical trials.

## Future Perspectives: Cetuximab And ICI

The efficacy of cetuximab has been partly attributed to its immunologic activity through ADCC, which is thought to link innate and adaptive antitumor immune responses via NK cells and antigen presenting cells that ultimately lead to EGFR-specific T cells (120, 121). Long-term survivorship described in patients with R/M HNSCC treated with cetuximab might be explained by sustained antitumor specific immune responses (122). The immunologic activity of cetuximab is of relevance in the era of immunotherapy. ICI will shortly become a backbone in the treatment of R/M HNSCC, and are already being investigated in the LA setting in combination with CRT or RT alone (NCT02952586, NCT03040999) (123, 124). Safety data from a phase I study combining ipilimumab (anti-CTLA-4 monoclonal antibody) with cetuximab and IMRT in LA-HNSCC (NCT01935921) was presented at ESMO meeting in 2016 by Bauman et al. (125). While dermatologic side-effects were the main dose-limiting toxicity of this combination, they were manageable, and treatment was felt to be overall well-tolerated. Results on efficacy are waiting. Growing evidence supports the investigation of antiPD-1/PD-L1 agents in combination with cetuximab and RT in LA-HNSCC (126, 127). The immunostimulatory effects attributed to RT, the increased antitumor immune infiltration induced by cetuximab and the blockade of inhibitory checkpoint receptors by ICI are hypothesized to act in a synergistic manner and ultimately revert the immune suppression of the HNSCC tumor microenvironment. As such, this triple combination is already being investigated in several clinical trials with different anti-PD-1/PD-L1 agents including avelumab (NCT02999087), durvalumab (NCT03051906) or nivolumab (NCT03349710) (128).

In R/M HNSCC disease, ICI are also being investigated in combination with cetuximab. Anti-PD-1, such as pembrolizumab or anti-PD-L1, such as avelumab in combination with cetuximab are being evaluated in phase II clinical trials [NCT03082534 and REACH study (NCT03082534), respectively]. Furthermore, preliminary data from an ongoing Phase I/II trial evaluating the safety and efficacy of the combination of monalizumab, a first-in-class monoclonal antibody targeting NK checkpoint receptor NKG2A, with cetuximab in previously treated R/M HNSCC patients reported increased response rates with the combination without potentiating the side effects of cetuximab (129).

Apart from ICI, other immunotherapies, such as motolimod (VTX-2337), a Toll-like receptor 8 agonist, are being investigated in combination with cetuximab (130) (NCT01836029). The addition of motolimod to the EXTREME regimen has been recently evaluated. Despite it was overall well-tolerated, it did not improve survival. However, in the subgroup analysis, patients with HPV-positive disease and those with injection site reactions seemed to benefit from the combination, suggesting that TLR8 stimulation may be useful in biomarker-selected patients (131).

Main clinical trials evaluating cetuximab combinations with ICI HNSCC are summarized on Table 3.

**Table 3:** Main clinical trials evaluating cetuximab combinations with ICI in HNSCC.

	***N***	**Treatment**	**Phase/status**	**Comments**
**LOCALLY-ADVANCED HNSCC**				
NCT02999087	688	Experimental arm: Avelumab + cetuximab + IMRT	Phase III/recruiting	Comparative arm: standard CRT with high dose cisplatin D1,22,43
NCT03349710	1046	Cohort 1: Experimental arm A: Nivolumab + cetuximab/placebo + IMRT Experimental arm B: Cetuximab + nivolumab/placebo + IMRT	Phase III/recruiting	Comparative double-blind, placebo-controlled, Phase 3 study. The study includes a 2nd cohort (cohort 2) with experimental arms C and D involving cisplatin + nivolumab/placebo
NCT03051906	69	Experimental arm: Cetuximab + durvalumab + IMRT followed by mainteinance durvalumab	Phase II/III/active, pending recruitment	Excludes oral cavity and HPV-positive oropharynx when T1-2, N0-N2a (AJCC, 7th ed.) or any T, any N with smoking history of <10 pack/years
NCT0193592	18	Experimental arm: Ipilimumab ± Cetuximab ± IMRT	Phase Ib/active, finished recruitment	Safety data presented at ESMO 2016 (125).
**R/M HNSCC**				
NCT02643550	100	Experimental arm: Monalizumab + cetuximab	Phase Ib-II	One arm for patients with prior exposure to PD-(L)1 ICI
EACH NCT03493322	130	Experimental arm: Avelumab + cetuximab Experimental arm: avelumab monotherapy	Phase II	
NCT03498378	24	Experimental arm: Avelumab + cetuximab + palbociclib	Phase I	
NCT00397384	83	Experimental arm: Pembrolizumab + cetuximab	Phase II	Four arms: cetuximab-naive, PD-(L)1-refractary-cetuximab-naive, PD-(L)1-refractary-cetuximab-refreactary and cutaneous HNSCC.
NCT01836029	175	Comparator arm: EXTREME Experimental arm: EXTREME + motolimod (VTX-2337, TLR8)	Phase II	Randomized

## Conclusions

Cetuximab is the only targeted therapy that has been proven effective for the treatment of HNSCC in both the LA and R/M settings. The incorporation of cetuximab not only expanded the range of treatment options in the past decade but also encouraged the investigation of many other targeted therapies in this tumor type. Particularly in LA-HNSCC, cetuximab has been crucial for the treatment of a subset of patients unfit for standard CRT due to baseline comorbidities or poor clinical condition. Despite this population was under-represented in the Bonner trial, RT-Cx has been the cornerstone in this subgroup of patients given its superiority when compared to RT alone. However, the lack of a direct comparison with CRT and the absence of predictive biomarkers of response to cetuximab have conditioned its widespread use in this setting. Results from the on-going clinical trials will hopefully shed light into this matter. In patients with HPV-positive OPC, the results from the RTOG-1016 and De-ESCALaTE phase III clinical trials have confirmed the inferiority of RT-Cx compared to standard CRT (cisplatin) in this disease, indicating that cetuximab is not an equivalent treatment option for de-escalation approaches in this patient population. The EXTREME regimen has remained the standard of care for the first line treatment of R/M-HNSCC in patients with PS 0–1. However, its use was not widespread likely due to the considerable toxicity and the logistics of managing 3 concomitant drugs including 5-FU. In the light of the recent results from the Keynote 048 study, the antiPD-1 agent pembrolizumab will likely become the new standard either alone or in combination with chemotherapy as first-line treatment for R/M HNSCC based on CPS PD-L1 expression. On-going trials evaluating cetuximab combinations with ICI and other immunotherapies might offer soon new treatment options in both LA and R/M HNSCC.

## Author Contributions

MT, MO, and RM: review concept, review design, interpretation, manuscript preparation, and manuscript review.

### Conflict of Interest Statement

RM has received personal fees and non-financial support from Merck, and personal fees from AstraZeneca, Nanobiotics, Bristol Myers, and MSD. MT has received non-financial support from Merck and Astra Zeneca, and personal fees from Merck, Nanobiotics, MSD, and Bristol Myers. Medical Oncology Department has received sponsorship for grants from Merck. MO has received personal fees from Merck.
