# Supplementary material for: Cetuximab-Containing Combinations in Locally Advanced and Recurrent or Metastatic Head and Neck Squamous Cell Carcinoma
Source: Front Oncol. 2019 May 20;9:383. doi: 10.3389/fonc.2019.00383 (PMC6536039; doi:10.3389/fonc.2019.00383)
Supplement: Supplementary file 1 [file Table_1.DOCX]

| **Supplementary Table 1: Phase II/III studies reported evaluating cetuximab combinations in LA-HNSCC** | | | | |
| --- | --- | --- | --- | --- |
| **Study**  **(Author, Year)** | **N** | **Treatment Arms** | **Phase** | **Primary Endpoint** |
| Bonner study  (Bonner, 2006) | 213 | Arm 1: RT  Arm 2: RT-Cx^1^  RT regimen/dose/fractions:   - Non-IMRT/70Gy in 35 Fractions - Non-IMRT/72-76.8 Gy in 60-64 fractions - Non-IMRT/ 72 Gy in 42 fractions | III | Duration of LRC |
| 05-003  (Argiris, 2010) | 39 | Single arm: ICT (docetaxel+carboplatin) + Cx^2^  followed by RT- cisplatin (30 mg/m^2^ weekly) + Cx^3^ and Cx^3^ maintenance for 6 months.  RT regimen/dose/fractions: RT (non-IMRT)/70Gy/35 fractions. | II | Objective response rate |
| 2003-0919  (Kies, 2010) | 47 | Single arm: ICT (weekly carboplatin+paclitaxel) – Cx^2^ followed by   - RT-cisplatin (100mg/m^2^ days 1,22) - RT alone - Surgery   Sequential treatment based on site/staging at diagnosis  RT regimen/dose/fractions IMRT and non-IMRT/66-72 Gy/30-42 fractions | II | Complete response rate to ICT |
| AlteRCC  (Merlano, 2011) | 45 | Single arm: RT-Cisplatin (20mg/m^2^ day1-5 week 1,4,7) – Cx^1^ (week 1-10)  RT regimen/dose/fractions: RT (non-IMRT)/ 66 Gy/ 30 fractions (day 1-5 weeks 2–3, 5–6 and 8–10) | II | Complete response rate |
| TREMPLIN  (Lefebvre, 2013) | 153 | ICT (TPF regimen): if response of primary tumor >50% randomized to:  Arm 1: RT-cisplatin (100mg/m^2^ days 1,22.43)  Arm 2: RT-Cx^1^  RT regimen/dose/fractions: RT (non-IMRT)/70Gy/35 fractions. | II | Larynx preservation rate at 3-months post-treatment |
| EMR-62202-717 study  (Strojan, 2013) | 30 | Single arm: ICT (TPF regimen) followed by RT-cisplatin (30mg/m^2^ weekly) – Cx^1^  RT regimen/dose/fractions: RT (non-IMRT)/70Gy/35 fractions. | II | LRC |
| RTOG 0522  (Ang, 2014) | 940 | Arm 1: RT-cisplatin (100mg/m^2^ days 1,22)  Arm 2: RT-cisplatin (100mg/m^2^ days 1,22) – Cx^1^  RT regimen/dose/fractions:   - Accelerated RT (non-IMRT)/72 Gy/42 fractions - IMRT-DAHANCA regimen/70 Gy/35 fractions | II | PFS |
| TTCC-01  (Mesia, 2015) | 50 | Single arm: ICT (TPF regimen) + Cx^2^ followed by RT-Cx^3^  RT regimen/dose/fractions: Accelerated-RT+concomitant boost/69.9 Gy/35 fractions | II | Objective response rate to ICT |
| CTXMAB+RT  (Magrini, 2016) | 70 | Arm 1: RT-Cx^1^  Arm 2: RT- cisplatin (weekly 40mg/m^2^)  RT regimen/dose/fractions: IMRT and non-IMRT/70 Gy/35 fractions | II | Treatment compliance and toxicity |
| 09-083  (Fury, 2016) | 30 | Single arm: RT-Cx^1^ – Cisplatin (50mg/m^2^ days 1,2,22,23) – Bevacizumab  RT regimen/dose/fractions: IMRT /70 Gy/35 fractions | II | 2-year PFS |
| 07-021 (Argiris, 2016) | 80 | Arm 1: RT-Cx^1^ – Pemetrexed  Arm 2: RT-Cx^1^ – Pemetrexed – Bevacizumab followed by maintenance Bevacizumab for 6 months.  RT regimen/dose/fractions: IMRT /70 Gy/35 fractions | II | 2-year PFS |
| EPIC HN  (Seiwert, 2016) | 110 | ICT (carboplatin+ weekly paclitaxel) + Cx^2^ followed by:  Arm 1: Hyperfractionated RT – 5Fu-hydroxiurea+ Cx^3^  Arm 2: Accelerated RT- Cisplatin- Cx^3^  RT regimen/dose/fractions: IMRT and non-IMRT/72 Gy/30-42 fractions. | II | PFS |
| TTCC-02  (Mesia, 2017) | 93 | Single arm: ICT (TPF regimen) followed by RT-Cx^1^ if response.  RT regimen/dose/fractions: IMRT and non-IMRT/70 Gy/35 fractions. | II | Rate of survival with functional larynx |
| E1308  (Marur, 2017) | 90 | ICT (cisplatin+paclitaxel) + Cx^2^  followed by:  Arm 1: low-dose IMRT (54Gy/27 fractions)- Cx ^3.^  Arm 2: standard-dose IMRT (69.3Gy/33 fractions - Cx^3^  Sequential treatment based on reponse to ICT  RT regimen/dose/fractions: IMRT /54-69.3 Gy/27-33 fractions | II | 2-year PFS |
| LCCC1103  (Weiss, 2018) | 40 | Single arm: ICT (Nab-paclitaxel+carboplatin) +Cx^2^ followed by:   - CRT (cisplatin 100mg/m^2^ days 1,22, 43 or 30mg/m^2^ weekly; cisplatin 20mg/m^2^ weekly plus weekly paclitaxel; weekly carboplatin +/- paclitaxel) - RT-Cx^3^   Concurrent treatment based on investigator and/or patient preferences or baseline comorbidities.  RT regimen/dose/fractions: IMRT/70 Gy/35 fractions. | II | Clinical response rate following ICT |
| GORTEC 2007-01  (Tao, 2018) | 406 | Arm 1: RT-Cx^1^ + Carboplatin 70mg/m² days 1, 22, 43 and 5FU 600 mg/m²/daily day 1-4, 22-25 and 43-46)  Arm 2: RT-Cx^1^  RT regimen/dose/fractions: IMRT/70 Gy/35 fractions | III | PFS |
| RTOG1016  (Gillison, 2018) | 987 | Arm 1: RT-cisplatin (100mg/m^2^ days 1,22)  Arm 2: RT-Cx^1^  RT regimen/dose/fractions: accelerated IMRT/ 70 Gy/ 35 fractions | III | OS |
| De-ESCALaTE (Mehanna, 2018) | 334 | Arm1: RT-cisplatin (100mg/m^2^ days 1,22,43)  Arm 2: RT-Cx^1^  RT regimen/dose/fractions: IMRT/70 Gy/35 fractions | III | Overall (acute and late) severe toxicity (grade 3-5) |

^1^Cx given at standard dose (leading 400mg/m^2^ week -1. followed by 250mg/m^2^ weekly concomitant to RT. ^2^Cx leading 400mg/m^2^ week 1 followed by 250mg/m^2^ weekly for the duration of the ICT regimen). ^3^ Cx 250mg/m^2^ weekly

Abbreviations: RT= radiotherapy; Cx= cetuximab; LRC= locoregional control; ICT= induction chemotherapy; PFS= progression-free survival; IMRT= intensity-modulated radiotherapy; OS= overall survival; CRT: chemoradiotherapy.
