# Supplementary material for: Cetuximab-Containing Combinations in Locally Advanced and Recurrent or Metastatic Head and Neck Squamous Cell Carcinoma
Source: Front Oncol. 2019 May 20;9:383. doi: 10.3389/fonc.2019.00383 (PMC6536039; doi:10.3389/fonc.2019.00383)
Supplement: Supplementary file 2 [file Table_2.DOCX]

| **Supplementary Table 2: Phase II/III studies reported evaluating cetuximab combinations in RM-HNSCC** | | | | |
| --- | --- | --- | --- | --- |
| **Study**  **(Author, Year)** | **N** | **Treatment Arms** | **Phase** | **Primary Endpoint** |
| EXTREME  (Vermorken JB, 2008) | 442 | Arm 1: cisplatin (100 mg/m2) or carboplatin (5AUC) and 5-FU (1000 mg/m2 per day for four days) every 3 weeks  Arm 2: cisplatin (100 mg/m2) or carboplatin (5AUC), 5-FU (1000 mg/m2 per day for four days) every 3 weeks and cetuximab (initial dose 400 mg/m2 and subsequent weekly 250 mg/m2)  -Maximum 6 cycles of chemotherapy in both arms  -Arm 2: patients who had at least stable disease received cetuximab monotherapy until the disease progressed or there were unacceptable toxic effects | III | OS |
| TTCC  (Hitt R, 2012) | 46 | Arm 1:  paclitaxel (80 mg/m2) and cetuximab (250 mg/m2; initial dose 400 mg/m2), weekly. | II | ORR |
|  |  |  |  |  |
| NCT01087970 (Vermorken JB,2013) | 66 | Arm 1: Cetuximab 250 mg/m2 (loading dose: 400mg/m2) days 1, 8 and 15; pemetrexed 500 mg/m2 +cisplatin 75 mg/m2 on day 1, every 3 weeks up to six cycle | II | PFS |
| NCT00409565 (Argiris A, 2013) | 46 | Arm 1: weekly cetuximab 250 mg/m2 (loading dose of 400 mg/m2) and bevacizumab 15 mg/kg ev día 1 on a 21 day cycle. | II | ORR |
| ADVANTAGE trial (Vermorken JB, 2014) | 182 | Arm 1 (EXTREME regimen): cisplatin (100 mg/m2) or carboplatin (5AUC), 5-FU (1000 mg/m2 per day for four days) every 3 weeks and cetuximab (initial dose 400 mg/m2 and subsequent weekly 250 mg/m2)  Arm 2: cisplatin (100 mg/m2) or carboplatin (5AUC), 5-FU (1000 mg/m2 per day for four days) every 3 weeks and cetuximab (initial dose 400 mg/m2 and subsequent weekly 250 mg/m2) AND cilengitide 500 mg days 1-4 and cilengitide 2000 mg days 8-15.  Arm 3: Arm 2: cisplatin (100 mg/m2) or carboplatin (5AUC), 5-FU (1000 mg/m2 per day for four days) every 3 weeks and cetuximab (initial dose 400 mg/m2 and subsequent weekly 250 mg/m2 AND cilengitide 2000 mg days 1,4,8,11,15 and 18  * Patients were treated for up to 6 cycles and then maintained with weekly cilengitide plus cetuximab (arms 2 and 3) or weekly cetuximab alone (arm 1) until disease progression or unacceptable toxicity | II | PFS |
| [NCT01252628](https://clinicaltrials.gov/show/NCT01252628)     (Jimeno, 2015) | 83 | Arm 1: weekly cetuximab 250 mg/m2 (loading dose of 400 mg/m2)  Arm 2:weekly cetuximab 250 mg/m2 (loading dose of 400 mg/m2) with PX-866 8 mg p.o. daily, a cycle of 21 days | II | PFS |
| TPEx (Guigay J, 2015) | 54 | Arm 1:  Docetaxel and cisplatin (75 mg/m2 both) at day 1 and weekly cetuximab 250 mg/m2 (loading dose of 400 mg/m2), repeated every 3 weeks for four cycles, followed by maintenance cetuximab 500 mg/m2 every 2 weeks | II | ORR after 4 cycles |
| B409  (Bossi P, 2017) | 201 | Arm 1: cisplatin (100 mg/m2) day 1/21 days, cetuximab (initial dose 400 mg/m2 and subsequent weekly 250 mg/m2)  Arm 2: : cisplatin (75 mg/m2) day 1/21 days, cetuximab (initial dose 400 mg/m2 and subsequent weekly 250 mg/m2) and paclitaxel 175 mg/m2 day 1/21 days.  *Maximum of 6 cycles of chemotherapy in both arms, cetuximab maintenance until disease progression or unacceptable toxicity. | IIb | PFS  (noninferiority) |
| Keynote 048 (Burtness B,  ESMO 2018) | 882 | Arm 1: Pembrolizumab 200 mg every 3 weeks  Arm 2: Pembrolizumab 200 mg + cisplatin (100 mg/m2) or carboplatin (5AUC), 5-FU (1000 mg/m2 per day for four days) (for 6 cycles every 3 weeks)  Arm 3: EXTREME regime | III | CPS ≥20, CPS≥1 and total population: -OS -PFS |

Abbreviations: OS= Overall survival; CPS= combined positive score, ORR= Overall response rate; PFS= progression-free survival.
